# Axitinib targets cardiac fibrosis in pressure overload-induced heart failure through VEGFA-KDR pathway

**DOI:** 10.3389/fmed.2023.1256156

**Published:** 2023-11-10

**Authors:** Tiantian Jiao, Yuanqi Wang, Xueqi Lin, Wei Song, Liang Wang, Tapu Md Sakibur Rahman, Linghao Xu, Lindong Nie, Qi Zhang, Jiming Li

**Affiliations:** ^1^Department of Cardiology, Shanghai East Hospital, Tongji University School of Medicine, Shanghai, China; ^2^Department of General Practice, Jinyang Community Health Service Center in Pudong District, Shanghai, China

**Keywords:** tyrosine kinase inhibitors, KDR, heart failure, cardiac fibrosis, axitinib

## Abstract

**Background:**

There are no specific clinical medications that target cardiac fibrosis in heart failure (HF). Recent studies have shown that tyrosine kinase inhibitors (TKIs) may benefit fibrosis in various organs. However, there is limited research on their application in cardiac fibrosis. Axitinib, an FDA-approved tyrosine kinase inhibitor, was used to evaluate its effects on cardiac fibrosis and function in pressure overload-induced heart failure.

**Methods:**

To build a pharmacological network, the pharmacological targets of axitinib were first retrieved from databases and coupled with key heart failure gene molecules for analysis and prediction. To validate the results outlined above, 8-week-old male C57BL/6 J mice were orally administrated of axitinib (30 mg/kg) daily for 8 weeks after Transverse Aortic Constriction (TAC) surgery. Mouse cardiomyocytes and cardiac fibroblasts were used as cell lines to test the function and mechanism of axitinib.

**Results:**

We found that the pharmacological targets of axitinib could form a pharmacological network with key genes involved in heart failure. The VEGFA-KDR pathway was found to be closely related to the differential gene expression of human heart-derived primary cardiomyocyte cell lines treated with axitinib, based on analysis of the publicly available dataset. The outcomes of animal experiments demonstrated that axitinib therapy greatly reduced cardiac fibrosis and improved TAC-induced cardiac dysfunction. Further research has shown that the expression of transforming growth factor-β(TGF-β) and other fibrosis genes was significantly reduced *in vivo* and *in vitro*.

**Conclusion:**

Our study provides evidence for the repurposing of axitinib to combat cardiac fibrosis, and offers new insights into the treatment of patients with HF.

## 1. Introduction

Heart failure (HF) is a complex syndrome that can result from various underlying causes, including coronary artery disease, hypertension, heart valve disorders, cardiomyopathies, and other cardiovascular conditions (1). HF is a prevalent and growing health problem worldwide. Despite advancements in drug and device therapy for heart failure, there has been limited progress in reducing hospitalization and mortality rates over the past decade (2). This emphasizes the urgent need to develop of novel therapies to effectively address the persistent challenges in managing heart failure.

Fibrosis, characterized by excessive accumulation of collagen and other extracellular matrix proteins in the heart, is a prominent pathological feature of heart failure (3). It occurs in response to ongoing injury and inflammation, leading to cardiac tissue remodeling. Various triggers, such as pressure overload, ischemia, or metabolic disturbances, initiate structural changes in the heart and activate fibroblasts to produce and deposit collagen fibers. Fibrosis can be a reparative mechanism that strengthens damaged myocardium (4). However, in chronic and progressive heart failure, fibrosis becomes excessive and detrimental, causing the myocardium to become stiff, compromising its ability to effectively pump blood (5). Moreover, fibrosis disrupts communication between cardiomyocytes, impairing their ability to contract synchronously and compromising overall cardiac performance (6). Despite recognizing the significance of cardiac fibrosis in HF, there remains an unmet medical need to effectively integrate its management into HF treatment (7).

Tyrosine kinase inhibitors (TKIs) are primarily used as anti-cancer medications. However, their therapeutic applications extend beyond oncology and have been explored for the treatment of various other conditions, including lung, liver, skin, and kidney fibrosis (8). Axitinib is a second-generation oral tyrosine kinase inhibitor (TKI) that received the Food and Drug Administration (FDA) approval in 2012 for the treatment of renal cell carcinomas (9). It is known for its potent inhibitory effects on the vascular endothelial growth factor (VEGF) receptor family, being approximately eight times more potent against these receptors than the platelet-derived growth factor (PDGF) receptor family (10). In our study, we explored the potential of axitinib to prevent adverse fibrotic remodeling in heart failure. By analyzing the pharmacological network of axitinib and heart fibrosis, we identified that the VEGFA-KDR pathway is closely associated with axitinib treatment. Our findings demonstrates that axitinib effectively reduced cardiac fibrosis, hypertrophy, and dysfunction induced by pressure overload in a mouse model.

## 2. Methods

### 2.1. Sequencing data acquisition and enrichment analyses

All the datasets used in this study were obtained from the GEO database.^1^ Dataset GSE133054 employs high-throughput sequencing to perform transcriptome sequencing of human cardiac tissues. We obtained transcriptome sequencing data from this dataset for patients without cardiomyopathy (NCM, *n* = 7) and with heart failure (HF, *n* = 7). Additionally, we acquired mRNA sequencing data for the human heart-derived primary cardiomyocyte cell lines control (*n* = 16) and cell lines treated with axitinib (*n* = 8) from the dataset GSE146096. For transcriptome data, we utilized Metascape^2^ for pathway enrichment and network analysis (11). Gene set enrichment analysis (GSEA) was performed using the clusterProfiler package (version 3.10.1) (12).

### 2.2. Pharmacological databases and Protein–Protein Interactions (PPI) analysis

The targets of axitinib were mainly obtained from the CTD^3^ database (13, 14) and the DGIbd database.^4^ To construct a PPI network from proteins of axitinib targets and enriched fibrosis-related pathways, the STRING^5^ database was employed. The PPI network was visualized using Cytoscape (version 3.10.0) for better interpretation and analysis (15). CytoHubba identified networks from a complex interactome (16).

### 2.3. Animal model

All animal experiments were reviewed and approved by the Ethics Committee of Tongji University. 8 weeks old male C57Bl/6 mice weighing ~20 g were purchased from the Shanghai Animal Administration Center. Mice were kept under a 12 h light/dark cycle and had free access to standard mouse chow and tap water. In our previous study, we established a mouse model of Transverse Aortic Constriction (TAC) surgery-induced heart failure (17). Axitinib (Selleck, USA) was dissolved in a solution of 0.5–1% Carboxymethylcellulose sodium (CMC). Oral gavage was used to administered at a dosage of 30 μg/g, once daily (qd). Axitinib administration commenced 1 day after surgery and continued for 8 weeks. The control group (NC) was treated with the same amount of CMC but without axitinib.

### 2.4. Echocardiography

The mice were anesthetized with isoflurane (2% isoflurane/O2 mixture). Parameters of cardiac morphology and function, including interventricular septal thickness, left ventricular internal diameter (LVID), left ventricular mass (LV mass corrected), ejection fraction (EF), and fractional shortening (FS), were examined using 2D M-mode and B-mode transthoracic echocardiography with Vevo2000 (VisualSonics, Canada).

### 2.5. Histological analysis

The mouse hearts were collected after euthanasia. For histological analysis, the heart tissues were fixed in 4% neutral formaldehyde at room temperature for more than 24 h. The specimens were fixed with formalin and embedded in paraffin, and then 4 μm thick serial sections were prepared along the short axis at the papillary muscle level. Slides were stained with Masson dye solution set (Servicebio, China) and wheat germ agglutinin (Servicebio, China) to examine fibrosis and cell size, respectively.

### 2.6. Quantitative real-time PCR

Total RNAs was extracted from animal and cell samples using TRIZOL reagent (Invitrogen, USA), and then reverse transcribed into cDNA using a reverse transcription kit (Vazyme Biotech, China). Quantitative real-time PCR (qRT-PCR) was performed using SYBR Green Master Mix (Vazyme, China). Specific primer pairs are listed in the following Table 1. The following PCR cycling conditions were used: 95°C for 3 min; 40 cycles of 95°C for 5 s and 60°C for 34 s; 95°C for 10 s; the melt curve, 65°C to 95°C, increment 0.5°C. Three biological replicates were used for each sample. Gene expression levels were analyzed using the 2^−△△Ct^ method with GAPDH as the reference gene.

**Table 1:** Primer table.

Gene	Forward primer	Reverse primer
mmu-GAPDH	GGTTGTCTCCTGCGACTTCA	TGGTCCAGGGTTTCTTACTCC
mmu-TGF-β	ACACATCAGAGCTCCGAGAA	GAGGTATCGCCAGGAATTGT
mmu-col-1	CTGGCGGTTCAGGTCCAAT	TTCCAGGCAATCCACGAGC
mmu-col-3	CCTGGCTCAAATGGCTCAC	CAGGACTGCCGTTATTCCCG
mmu-ACTA2	CCCAGACATCAGGGAGTAATGG	TCTATCGGATACTTCAGCGTCA
mmu-MAPK14	ACCTAGCTGTGAACGAAGACT	GTAGCCACGTAGCCTGTCATC
mmu-PRKCA	GTTTACCCGGCCAACGACT	GGGCGATGAATTTGTGGTCTT

### 2.7. Cell culture

The cell bank of the Shanghai Branch of the Chinese Academy of Sciences provided mouse cardiomyocytes (MCM) and mouse cardiac fibroblasts (MCF), which were cultured in DMEM/F12 medium (Servicebio, China) supplemented with 10% fetal bovine serum (Gibco, USA) and 100 g/mL penicillin/streptomycin. The culture medium was replaced every 2/3 days. Angiotensin II (Ang II, 1 μM) treatment was performed on the MCM for 48 h to induce myocardial hypertrophy. MCF were treated with transforming growth factor-β (TGF-β, 10 ng/mL) for 1 h to induce fibrosis. Axitinib was dissolved in dimethyl sulfoxide (DMSO) and treated with MCM and MCF at 1 μM for 24 h.

### 2.8. Statistical analysis

Descriptive statistics were used to summarize the clinical characteristics of patients. Quantitative data are presented as mean ± Standard Deviation (SD). Statistical significance was assessed through t-tests or one-way analysis of variance (ANOVA) for sample sizes exceeding 6. For smaller sample sizes, the Mann–Whitney U test was employed for two groups, while the Kruskal-Wallis test, followed by Dunn’s *post hoc* test, was utilized for scenarios involving three or more groups. For biological replicates, the independent procedures were repeated three times. Charting and statistical analyses were performed using GraphPad Prism 5.0 software (GraphPad, USA). A *p* value <0.05 was considered as statistically significant (*p < 0.05, **p < 0.01, and ***p < 0.001).

## 3. Results

### 3.1. Significant enrichment of the fibrosis pathway in transcriptomic sequencing analysis of heart failure

**Figure 1:**
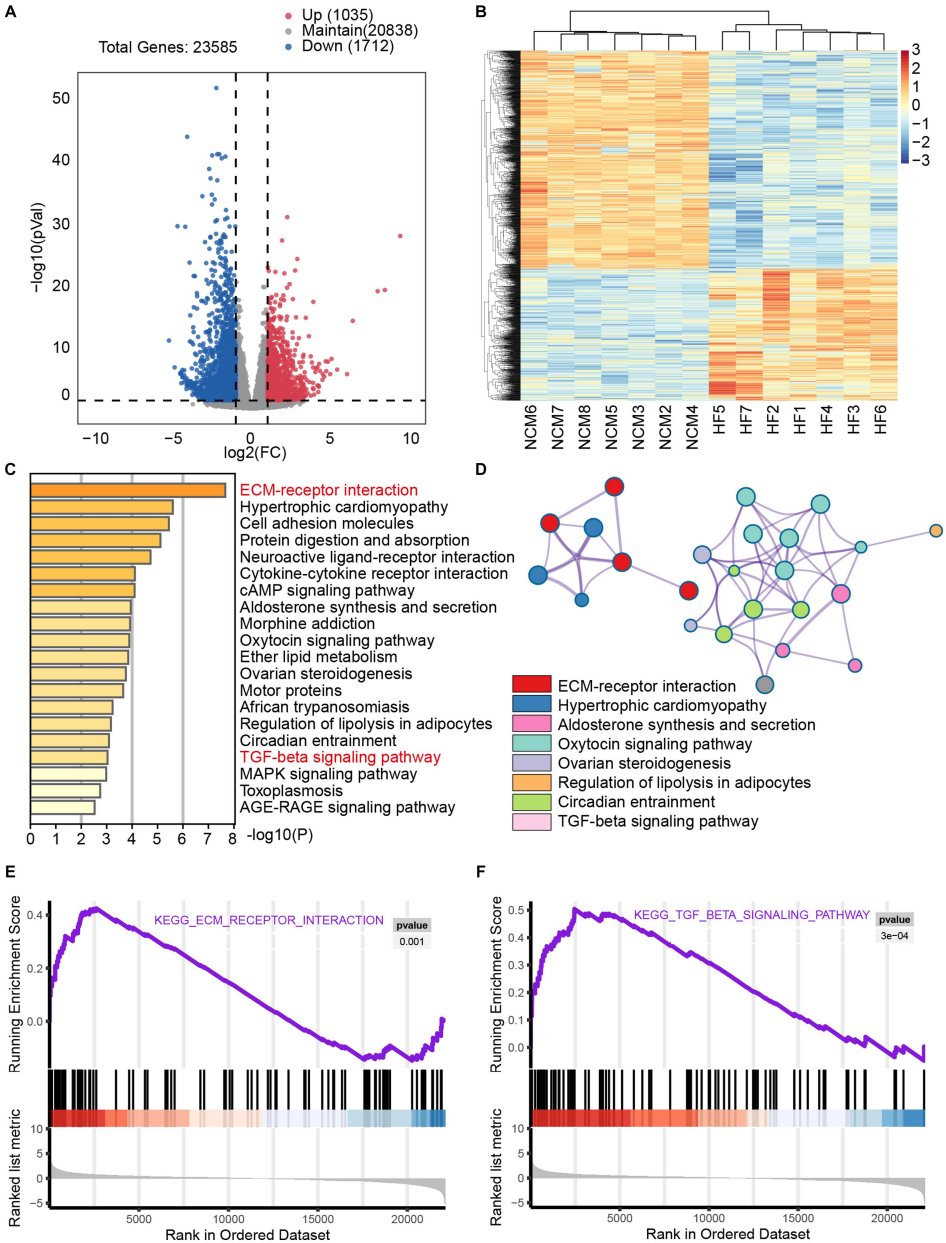
Significant enrichment of the fibrosis pathway in transcriptomic sequencing analysis of heart failure The dataset GSE133054 consists of 8 samples of non-cardiomyopathy and 7 samples of human heart failure tissue. **(A)** Volcano plot provides a graphical representation of the distribution of gene fold change (FC) and *p*-values. This plot is commonly used to visualize the results of differential analysis, where downregulated genes are depicted in blue and upregulated genes in red, highlighting significant changes. **(B)** The heat map was used to visually display the expression differences of the selected upregulated and downregulated genes from the volcano plot between the two groups of populations. In the heatmap, blue represents significantly downregulated genes, whereas red represents significantly upregulated genes. **(C)** Metascape analysis showed significantly enriched pathways including myocardial hypertrophy, fibrosis, and heart failure. **(D)** Network of enriched terms colored by cluster ID, where nodes that share the same cluster ID are typically close to each other. Additionally, GSEA analysis of the ECM-receptor interaction pathway **(E)** and TGF-β pathway **(F)** revealed significant enrichment of fibrosis pathways in patients with heart failure.

To investigate the impact of fibrosis in heart failure, we examined the GSE133054 dataset, comprising 8 samples of normal tissue and 7 samples of heart failure tissue. mRNA sequencing identified 23,585 genes, and differential gene expression analysis using the established heart failure criteria (*p* < 0.05, absolute fold change >2) revealed 2,747 differentially expressed genes, with 1,035 upregulated and 1,712 downregulated genes (Figures 1A,B). To elucidate the biological functions of the differentially expressed genes, we conducted pathway analysis using Metascape. Further analysis using Metascape for KEGG pathway enrichment reveal notable enrichment of the ECM-receptor interaction pathway, as well as hypertrophic cardiomyopathy and cell adhesion molecules. In addition, the TGF-β pathway was significant enriched (Figures 1C,D). KEGG differential gene enrichment analysis relies on identifying significantly upregulated or downregulated genes, which may result in overlooking genes that hold significant biological significance, but do not show significant differential expression. In contrast, gene set enrichment analysis (GSEA) does not depend on a specific threshold for differential gene expression. The GSEA algorithm evaluates the overall trend and considers the enrichment of gene sets, thereby increasing the likelihood of capturing subtle yet coordinated changes that influence biological pathways. GSEA analysis provided additional confirmation of the significant enrichment of the TGF-β pathway and the ECM-receptor interaction pathway (Figures 1E,F). Supplementary Figures 1, 2 present detailed information on the TGF-βand ECM-receptor interaction pathways. The extracellular matrix (ECM) signaling pathway operates utilizes ECM receptors, primarily integrins, to recruit and activate effector cells that promote fibrosis. TGF-β signaling is one of the most important signaling pathways required for cardiac fibrosis. We gathered key differentially expressed genes associated with ECM-receptor interactions and the TGF-β pathway to facilitate subsequent analysis. Collectively, these findings underscore the critical role of fibrosis-related pathways in human HF progression.

### 3.2. Axitinib targets fibrosis-related genes based on the pharmacological protein–protein interaction (PPI) network

**Figure 2:**
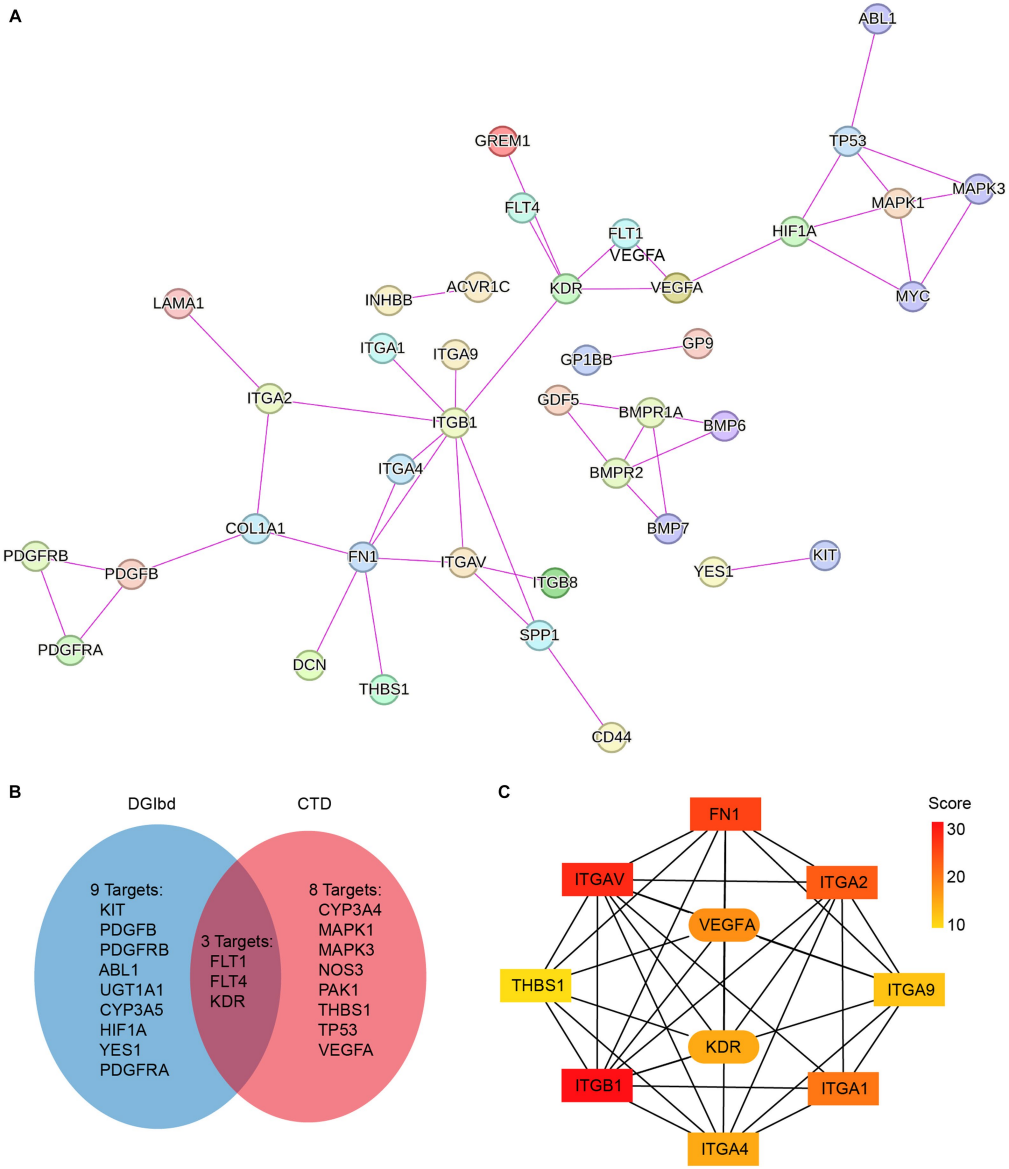
Pharmacological databases constructing PPI network of axitinib target genes and fibrosis-related genes. **(A)** Axitinib target genes were integrated with key differentially expressed genes that are associated with ECM-receptor interaction and TGF-β pathway, and a protein–protein interaction (PPI) network was constructed using String. **(B)** Venn diagram showed axitinib target genes that were gathered in two pharmacological databases, CTD and DGIbd. **(C)** Using CytoHubba in Cytoscape, the top 10 genes in the STRING network were identified and ranked using the MNC method. VEGFA-KDR exhibits a particularly strong correlation with fibrosis-related genes among the target genes of axitinib.

To investigate the pharmacological effects of axitinib, we analyzed its target genes using CTD (see footnote 3) and DGIbd (see footnote 4). The CTD is a robust, publicly available database that aims to advance the understanding of how environmental exposures affect human health. It provides manually curated information about chemical-gene/protein interactions, chemical-disease and gene-disease relationships. The drug-gene interaction database (DGIdb) mined drug-gene interactions from DrugBank, PharmGKB, Chembl, Drug Target Commons, TTD, and others, using a combination of expert curation and text-mining. A total of 20 target genes for axitinib were identified (Figure 2B). These axitinib target genes were then integrated with key differentially expressed genes associated with ECM-receptor interaction and the TGF-β pathway, and a protein–protein interaction (PPI) network was constructed using STRING (Figure 2A). Using CytoHubba, we identified key nodes in the network and discovered that VEGFA and VEGFR2 (KDR) were closely associated with eight fibrosis-related genes, namely FN1, ITGA1, ITGA2, ITGA4, ITGA9, ITGAV, ITGB1, and THBS1 (Figure 2C).

### 3.3. Axitinib predominantly modulates the VEGFA-KDR pathway in cardiomyocyte

**Figure 3:**
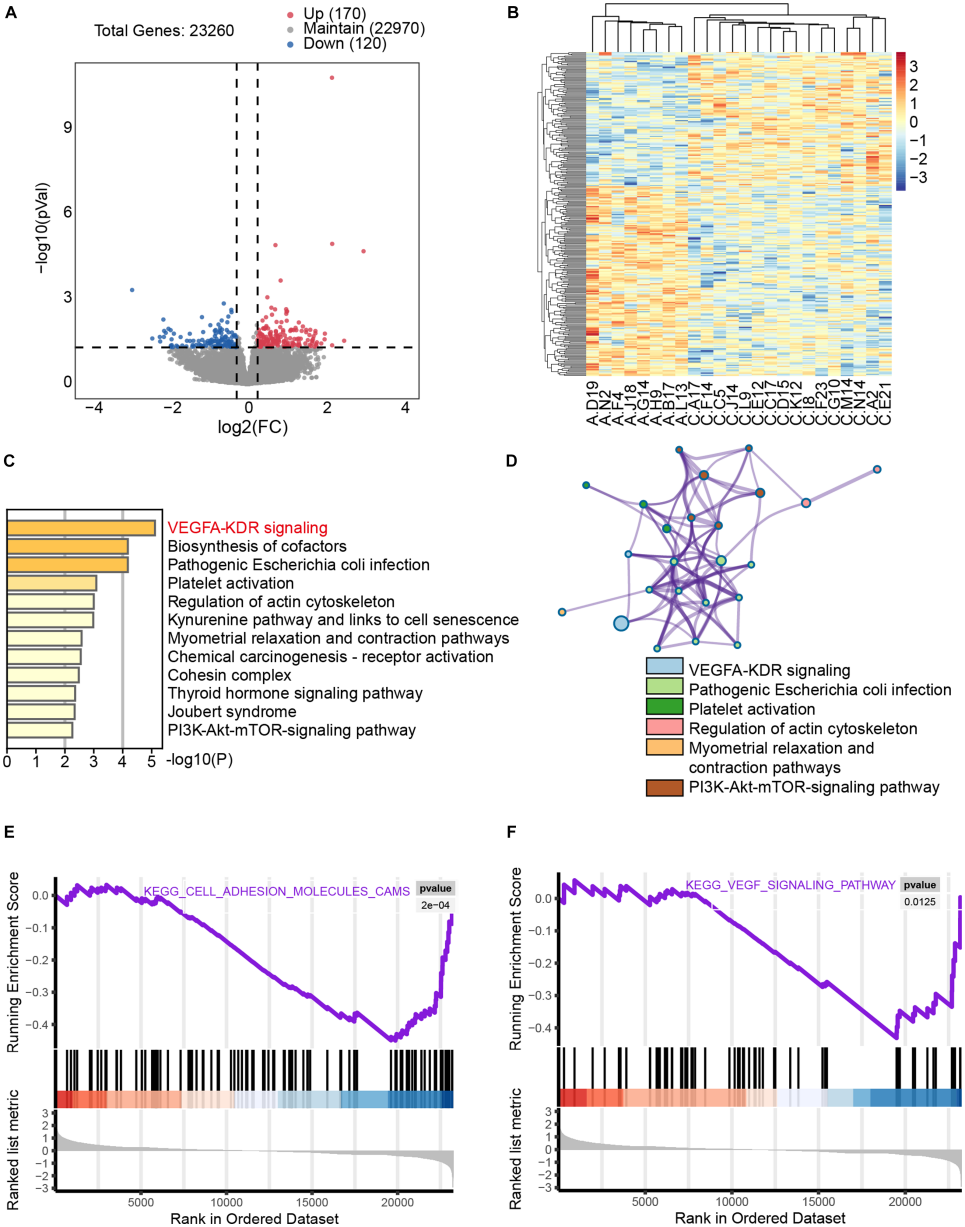
Significant enrichment of the VEGFA-KDR pathway in axitinib-treated cardiac myocytes (iPSC-CM) mRNA sequencing data for control iPSC-CMs (*n* = 16) and iPSC-CMs treated with axitinib (*n* = 8) from dataset GSE146096. **(A)** Volcano plot of transcriptomic analysis of axitinib-treated cardiac myocytes (iPSC-CMs). **(B)** Heatmap of differentially expressed genes in axitinib-treated cardiac myocytes (iPSC-CMs). Volcano plot and heatmap were used to visually display the gene expression differences. Blue represents significantly downregulated genes, whereas red represents significantly upregulated genes. **(C,D)** KEGG enriched pathways in axitinib-treated cardiac myocytes (iPSC-CMs) identified by Metascape analysis indicated a significant enrichment of the VEGFA-KDR signaling pathway. Moreover, GSEA analysis of the cell adhesion molecule pathway **(E)** and VEGF signaling pathway **(F)** was consistent with the KEGG enriched pathways.

To investigate the effects of axitinib on cardiac myocytes, we analyzed the GSE146096 dataset, which includes 16 control human induced pluripotent stem cell-derived cardiomyocytes (iPSC-CMs) cells and 8 iPSC-CMs treated with axitinib for 48 h. Applying a significance cutoff of *p* < 0.05 and an absolute fold change greater than 1.2, we identified 290 differentially expressed genes, with 170 upregulated and 120 downregulated genes (Figures 3A,B). Metascape analysis demonstrated significant enrichment of the VEGFA-KDR pathway (Figures 3C,D). Additionally, GSEA confirmed the significant enrichment of cell adhesion and VEGF signaling pathways (Figures 3E,F). These findings suggest that the VEGFA-KDR pathway is significantly affected in axitinib-treated cardiac myocytes. Consequently, we propose that axitinib may influence the progression of cardiac fibrosis through its involvement in the VEGFA-KDR pathway.

### 3.4. The effects of axitinib on cardiac fibrosis *in vivo* and *in vitro*

**Figure 4:**
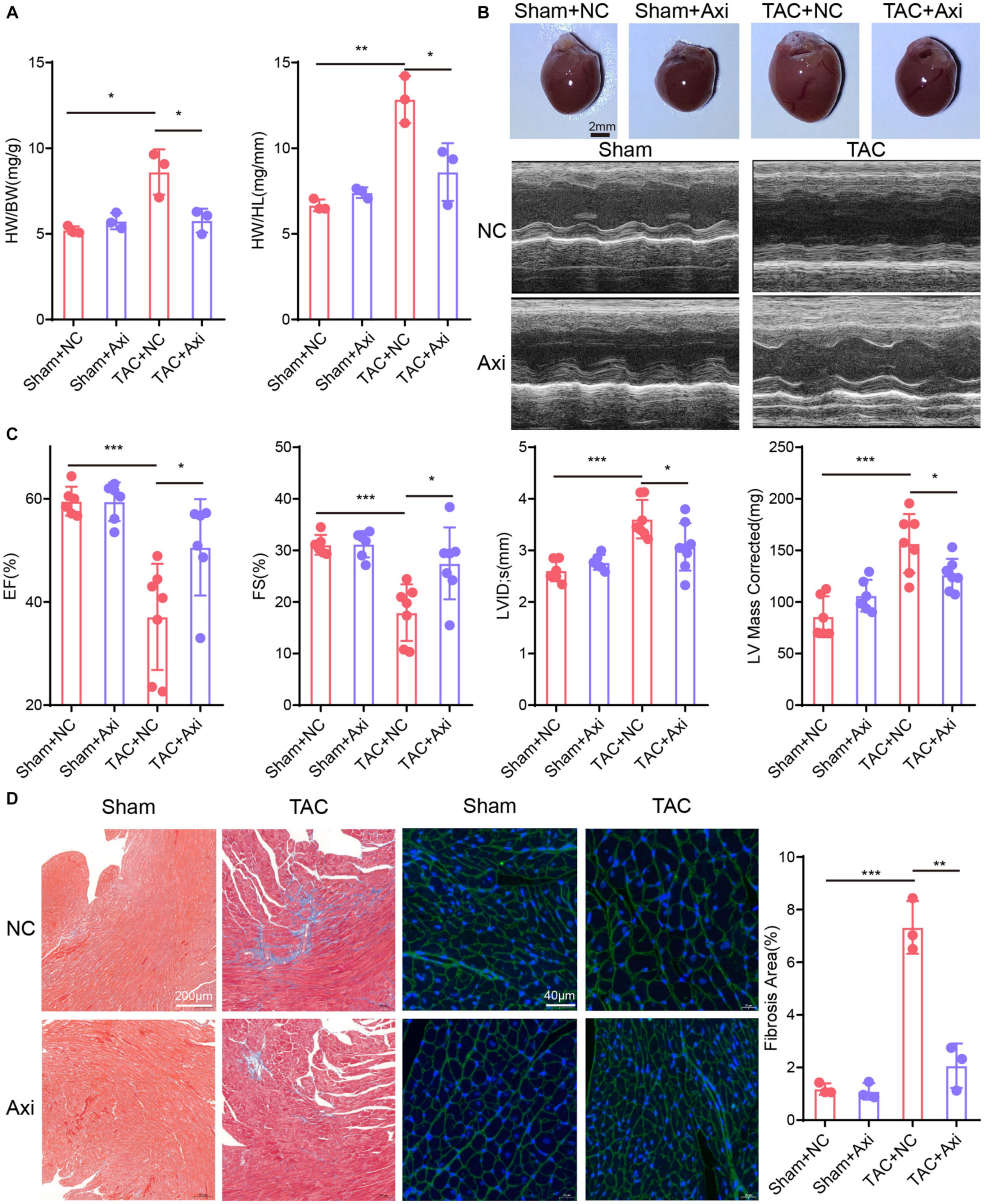
Axitinib treatment improves TAC-induced heart failure and cardiac fibrosis. TAC surgery was performed on 8-week-old C57BL/6 J mice and axitinib was administered. The mice were divided into four groups: Sham+NC, Sham+Axi, TAC + NC and TAC + Axi. **(A)** Heart weight/body weight (HW/BW) and heart weight/tibia length (HW/HL) increased after TAC surgery and decreased after axitinib treatment (*n* = 3 in each group). **(B)** Representative cardiac specimens and echocardiographic images were shown for visual display. Scale Bar = 2 mm. **(C)** Cardiac ultrasound results showed that axitinib protected cardiac function from pressure overload-induced heart failure (*n* ≥ 6 in each group). **(D)** Masson’s trichrome (Scale Bar = 200 μm) and WGA staining (Scale Bar = 40 μm) with bar graphs revealed decreased collagen deposition and smaller cardiomyocytes in the hearts of the axitinib-treated group. HW/BW, heart weight/ body weight; HW/HL, heart weight/tibia length; LVEF or EF, left ventricular ejection fraction; LVFS or FS, left ventricular fractional shortening; LVID, left ventricular internal diameter; LV mass corrected, left ventricular mass; *p < 0.05, **p < 0.01, and ***p < 0.001.

We assessed the therapeutic efficacy of axitinib in a well-established mouse model of heart failure induced by transverse aortic constriction (TAC). Our results confirmed the successful establishment of a TAC-induced cardiac pathological remodeling mouse model for changes in heart size and cardiac function (Figures 4A–C). TAC surgery was performed on 8-week-old C57BL/6 J mice, and axitinib was administered throughout the experimental period to investigate its effect on pressure overload-induced cardiac pathology. Mice were divided into four groups: Sham+NC, Sham+axitinib, TAC + NC, and TAC + axitinib according to their surgery and treatment. TAC mice treated with axitinib exhibited a lower heart weight/ body weight (HW/BW) ratio and lower heart weight/tibia length (HW/HL) ratio than those in TAC + NC group (Figure 4A). The axitinib-treated group demonstrated a significant improvement in the left ventricular ejection fraction (LVEF) and left ventricular fractional shortening (LVFS) compared to the NC group after TAC surgery, indicating enhanced contractile function. Additionally, the axitinib-treated group exhibited reduced left ventricular internal diameter (LVID), left ventricular mass (LV mass corrected), and heart weight, suggesting that axitinib treatment ameliorated cardiac function and prevented adverse cardiac remodeling (Figure 4C). Representative cardiac specimens and echocardiographic images are shown in Figure 4B, which are consistent with the results in Figures 4A,C. To evaluate cardiac fibrosis after TAC surgery, Masson and WGA staining were performed, revealing decreased collagen deposition and smaller cardiomyocytes in the hearts of the axitinib-treated group (Figure 4D).

**Figure 5:**
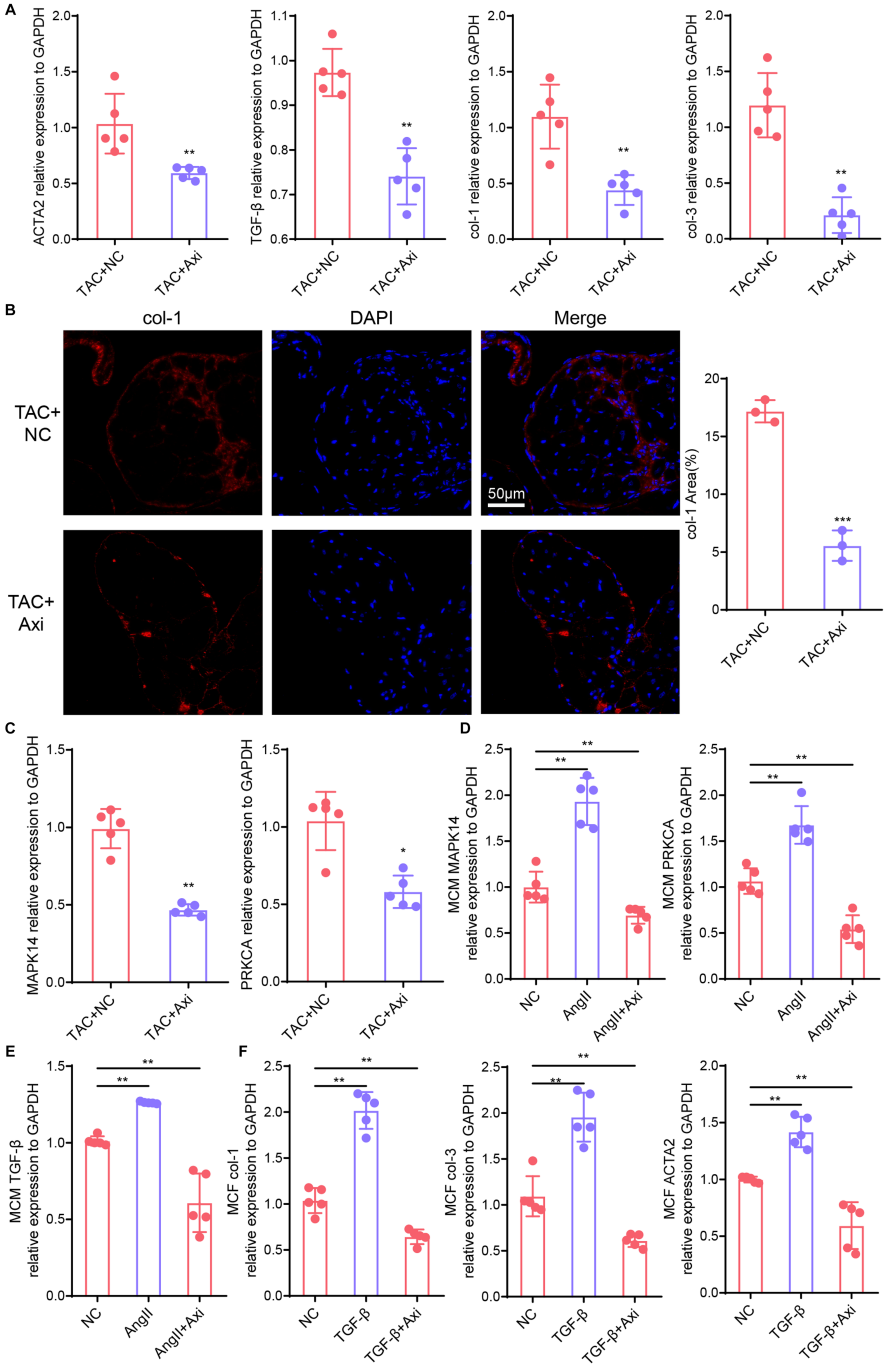
Axitinib treatment improves the expression of fibrosis markers. **(A)** The expression levels of genes associated with cardiac fibrosis (TGF-β, ACTA2, col-1 and col-3) were significantly reduced in the axitinib-treated TAC mice by qPCR compared with TAC mice(*n* = 5). **(B)** Immunohistochemical staining results for collagen-1 (col-1) in axitinib-treated TAC mice. **(C)** Expression of MAPK14 and PRKCA were changed in axitinib-treated TAC mice compared with TAC mice (*n* = 5). Next, we stimulated cardiomyocytes with AngII and mouse cardiac fibroblasts with TGF-β to induce hypertrophy and fibrosis. **(D)** Expression of MAPK14 and PRKCA in axitinib-treated mouse cardiomyocytes stimulated with AngII showed similar changes *in vivo* (*n* = 5). Axitinib treatment inhibited TGF-β expression in cardiomyocytes **(E)** and fibrosis-associated genes (col-1, col-3 and ACTA2) in mouse cardiac fibroblast **(F)** (*n* = 5). TGF-β, transforming growth factor-β; col-1, collagen type I; col-3, collagen type III; ACTA2, actin alpha 2; MAPK14, mitogen-activated protein kinase 14; PRKCA, protein kinase C alpha; Ang II, angiotensin II; *p < 0.05, **p < 0.01, and ***p < 0.001.

Furthermore, we examined fibrosis markers in the hearts of axitinib-treated and NC groups in the TAC model. qPCR showed that the expression levels of genes associated with cardiac fibrosis (TGF-β, ACTA2, col-1 and col-3) were significantly reduced in axitinib-treated TAC mice (Figure 5A). The axitinib-treated group exhibited significantly increased expression of collagen-I in the heart tissue (Figure 5B), indicating an improvement in pathological cardiac remodeling. We then explored the expression of downstream VEGF signaling pathway, such as MAPK14 and PRKCA, which were altered in axitinib-treated TAC mice (Figure 5C). Mouse cardiomyocytes and cardiac fibroblasts were used as cell lines to test the function and mechanism of axitinib *in vitro*. Axitinib-treated mouse cardiomyocytes showed similar changes in the downstream VEGF signaling pathway when stimulated by AngII (Figure 5D). Subsequently, we stimulated cardiomyocytes with AngII and mouse cardiac fibroblasts with TGF-β. The results demonstrated that axitinib treatment inhibited TGF-β expression in cardiomyocytes and fibrosis-associated genes in mouse cardiac fibroblasts (Figures 5E,F). These findings suggested that axitinib exerts inhibitory effects on cardiac fibrosis in both *in vivo* and *in vitro* mouse models.

## 4. Discussion

Myocardial fibrosis, a variety of pathological factors caused by cardiomyocytes and myocardial interstitial changes that cause cardiac systolic and diastolic dysfunction, myocardial function and metabolism abnormalities, is a common cardiovascular disease occurrence and development to a certain stage of the main pathological manifestations (18). Our study, based on transcriptomic sequencing analysis of human heart failure, showed that the fibrosis pathway was significantly enriched. This is consistent with previous published work in human studies (19, 20). Fibrosis is initiated by myocardial injury and sterile myocardial inflammation in HF (6, 21). Cardiac fibrosis is characterized by excessive deposition of ECM proteins, which are influenced by various profibrotic stimuli, including TGF-β, Ang II, and aldosterone (21). The fibrotic response involves myofibroblasts, which directly produce fibrous tissue, and cardiomyocytes that indirectly contribute to fibrosis by secreting profibrotic mediators (3). Despite the use of conventional therapies, such as RAAS inhibitors, to reduce myocardial fibrosis in patients with HF (22–24), fibrosis remains unresolved, highlighting the need to explore novel treatments.

Progress has been made in the treatment of fibrosis in other organs, such as the lungs, liver, and kidneys. Pirfenidone has been approved by FDA for the treatment of idiopathic pulmonary fibrosis (IPF), which is known to reduce disease progression and improve lung function, exercise tolerance, and progression-free survival in patients with IPF (25–27). Another drug, nintedanib, is approved for the treatment of lung fibrosis in patients with IPF (28–30). An increasing number of studies have explored the potential of pirfenidone and nintedanib in liver and kidney fibrosis (31). In heart failure, pirfenidone may reduce the expression of col-1 induced by TGFβ, decrease vascular permeability, and inhibit inflammation and fibrosis caused by NLRP3, thereby alleviating the development of pressure overload-induced chronic cardiac fibrosis (32, 33). Furthermore, nintedanib demonstrated favorable outcomes in pressure overload-induced cardiac remodeling and dysfunction by engaging multiple interrelated mechanisms that directly impact immune cells, cardiac fibroblasts, and cardiomyocytes (34). Nintedanib is a tyrosine kinase inhibitor with a broad spectrum of targets including PDGFR, FGFR, and VEGF. Many in-depth studies have been conducted on the molecular mechanisms of the pathogenesis of cardiac fibrosis, and based on these mechanisms, a variety of molecular therapeutic targets have been identified PDGF and VEGF, which are the most important growth factors involved in fibrin synthesis (35, 36). As a developed multi-target tyrosine kinase inhibitor, axitinib selectively targets VEGFR (Flt-1, KDR and Flt-4) and has shown stronger inhibitory effects in early clinical trials. Axitinib exhibits approximately eight-fold higher potency against the VEGF receptor family than the PDGF receptor family (10). Axitinib has more efficient anti-fibrosis potential and prospects for clinical application (37–39). However, no studies evaluated the efficacy of axitinib for the treatment of cardiac fibrosis. To investigate the potential therapeutic effects of axitinib on cardiac fibrosis, we initially collected pharmacological targets of axitinib from two distinct pharmacological databases. Subsequently, we identified key genes associated with heart failure through significant enrichment of the fibrosis pathway in transcriptomic sequencing analysis. Using the STRING database, we constructed a PPI network that revealed a strong correlation between axitinib and cardiac fibrosis.

Studies have shown that cardiac fibrosis is closely associated with cardiomyocytes and myocardial fibroblasts (18, 21). Cardiomyocytes are the primary cellular components of the heart, accounting for 49.2% of the ventricular mass and making them the predominant cell type (40). Emerging evidence indicates that functional cardiomyocytes have the potential to stimulate interstitial fibroblasts during periods of stress, thereby promoting the development of interstitial fibrosis (41). However, it is still unclear whether cardiomyocytes respond to axitinib treatment and how this may impact cardiac fibrosis. By analyzing the sequencing data of iPSC-CM cells treated with axitinib, we found that the VEGFA-KDR pathway was activated. Previous studies have shown that the VEGFA-KDR pathway is closely related to heart failure (42, 43). In the cardiac fibrosis model afflicted by disease, there was a reduction in vascular endothelial growth factor (VEGF) when compared to non-diseased controls, while its receptors, KDR, exhibited an upregulation (44–46).

TKIs have been associated with a growing number of cardiovascular side effects. A meta-analysis of clinical trials involving 10,647 patients treated with various TKIs, including axitinib, pazopanib, sorafenib, sunitinib, and vandetanib, for different types of malignancies demonstrated an overall incidence of asymptomatic left ventricular systolic dysfunction of 2.4%, with 1.2% developing symptomatic heart failure (47). These side effects of TKIs can be attributed to both the “on-target” effects of VEGF inhibition and the “off-target” effects resulting from the inhibition of other tyrosine kinases (48). Additionally, cardiac dysfunction associated with TKIs is partially reversible. In a randomized controlled trial of sunitinib for imatinib-resistant gastrointestinal stromal cancer, all patients who developed heart failure showed improvement in EF and resolution of symptoms after dose adjustment and heart failure therapy (49). Endomyocardial biopsy of two patients with heart failure revealed cardiomyocyte hypertrophy and abnormal mitochondrial configurations, but no evidence of apoptosis or fibrosis (49).

Research on the role of axitinib in heart failure is limited and insufficient. The exact correlation between TKIs and the development of cardiac dysfunction remains unknown because preclinical studies examining potential toxicities are usually performed in the absence of cancer (48). Similar results have been observed in pulmonary fibrosis. Of the 28 TKIs currently approved, 16 (57%) have been reported to induce interstitial lung disease (ILD) with varying frequency and/or severity (50, 51). However, Animal models have demonstrated that several tyrosine kinase inhibitors such as imatinib, nilotinib, and gefitinib can effectively reduce pulmonary fibrosis (51). To investigate the anti-fibrotic effects of axitinib, we administered axitinib to a mouse model of pressure overload-induced heart failure and assessed its impact on pathological remodeling and cardiac dysfunction. We found that axitinib prevented myocardial dysfunction, fibrotic remodeling and pathological hypertrophy induced by pressure overload in mice. Combined with the results of our bioinformatics analysis, which showed that axitinib led to significant enrichment of the VEGFA-KDR pathway in cardiomyocytes, this effect may be attributed to the inhibition of key signaling pathways involved in fibrosis progression. TGF-β plays a crucial role in fibrosis by promoting the production of collagen and other extracellular matrix proteins. Our cell experiment results showed that axitinib inhibited TGF-β signaling, leading to reduced collagen deposition and fibrotic changes in heart tissue. The results suggested to us axitinib exerted anti-fibrotic effects through the suppression of factors such as transforming growth factor-beta (TGF-β) and its downstream signaling molecules and this inhibition of TGF-β signaling by axitinib might occur through the modulation of its receptor, KDR, which is involved in TGF-β signaling pathways (52). Since we observed a reduction in cardiac hypertrophy in axitinib-treated animals, we were interested in determining whether axitinib directly affects cardiomyocytes through anti-fibrotic mechanisms. Our experimental results showed significant alterations in the key signaling pathways underlying fibrosis. The expression of TGF-β in cardiomyocytes significantly increased after drug treatment. Myocardial fibroblasts activated with TGF-β showed a significant reduction in fibrosis markers after drug treatment.

This study exhibits several limitations. Firstly, the mice in this investigation received Axitinib treatment for 8 weeks following TAC surgery, yet the assessment did not encompass the long-term consequences of this treatment. Evaluating the long-term effectiveness and safety of this approach is imperative for its clinical application. Secondly, the study did not integrate interventions aimed at modifying the VEGFA-KDR pathway to mitigate the effects of Axitinib. Further experiments are needed to validate these findings in future research. Additionally, the absence of human research data is notable. Human studies are typically more convincing when considering drug repurposing.

In summary, this study preliminarily verified that axitinib plays an anti-fibrotic role by inhibiting the TGF-β signaling pathway of cardiomyocytes and myocardial fibroblasts in cardiac tissue. This lays a foundation for the clinical application of axitinib in the treatment of cardiac fibrosis. However, further studies are warranted to delineate the detailed mechanisms of axitinib therapeutic efficacy and define the contribution of various cells, including fibroblasts and cardiomyocytes.

## Data availability statement

Publicly available datasets were analyzed in this study. This data can be found at: GEO database (GSE133054 and GSE146096).

## Ethics statement

The animal study was approved by the Ethics Committee of Tongji University. The study was conducted in accordance with the local legislation and institutional requirements. All animal experiments were reviewed and approved by the Ethics Committee of Tongji University.

## Author contributions

TJ: Conceptualization, Methodology, Writing – original draft. YW: Conceptualization, Methodology, Writing – review & editing. XL: Formal analysis, Validation, Writing – review & editing. WS: Data curation, Validation, Writing – original draft. LW: Data curation, Software, Validation, Writing – original draft. TR: Data curation, Formal analysis, Writing – review & editing. LX: Formal analysis, Visualization, Writing – original draft. LN: Investigation, Software, Writing – original draft. QZ: Conceptualization, Supervision, Writing – review & editing. JL: Conceptualization, Funding acquisition, Supervision, Writing – review & editing.

## Footnotes

^1^https://www.ncbi.nlm.nih.gov/geo/

^2^https://www.metascape.org/gp/index.html

^3^http://ctdbase.org/

^4^https://www.dgidb.org/

^5^http://version-12-0.string-db.org

## References

[1] Fudim M Ali-Ahmed F Parzynski CS Ambrosy AP Friedman DJ Pokorney SD. Periprocedural risk and survival associated with implantable cardioverter-defibrillator placement in older patients with advanced heart failure. JAMA Cardiol. (2020) 5:643–51. doi: 10.1001/jamacardio.2020.0391, PMID: 32211811 PMC7097837

[2] Bansal N Zelnick L Bhat Z Dobre M He J Lash J. Burden and outcomes of heart failure hospitalizations in adults with chronic kidney disease. J Am Coll Cardiol. (2019) 73:2691–700. doi: 10.1016/j.jacc.2019.02.071, PMID: 31146814 PMC6590908

[3] Gonzalez A Schelbert EB Diez J Butler J. Myocardial interstitial fibrosis in heart failure: biological and translational perspectives. J Am Coll Cardiol. (2018) 71:1696–706. doi: 10.1016/j.jacc.2018.02.021 29650126

[4] Seo HH Lee S Lee CY Lee J Shin S Song BW. Multipoint targeting of Tgf-Beta/Wnt transactivation circuit with Microrna 384-5p for cardiac fibrosis. Cell Death Differ. (2019) 26:1107–23. doi: 10.1038/s41418-018-0187-3, PMID: 30206318 PMC6748152

[5] Stratton MS Bagchi RA Felisbino MB Hirsch RA Smith HE Riching AS. Dynamic chromatin targeting of Brd4 stimulates cardiac fibroblast activation. Circ Res. (2019) 125:662–77. doi: 10.1161/CIRCRESAHA.119.315125, PMID: 31409188 PMC7310347

[6] Bacmeister L Schwarzl M Warnke S Stoffers B Blankenberg S Westermann D. Inflammation and fibrosis in murine models of heart failure. Basic Res Cardiol. (2019) 114:19. doi: 10.1007/s00395-019-0722-5 30887214

[7] Diez J de Boer RA. Management of Cardiac Fibrosis is the largest unmet medical need in heart failure. Cardiovasc Res. (2022) 118:e20–2. doi: 10.1093/cvr/cvab228, PMID: 34244741

[8] Heldin CH Lennartsson J Westermark B. Involvement of platelet-derived growth factor ligands and receptors in tumorigenesis. J Intern Med. (2018) 283:16–44. doi: 10.1111/joim.12690, PMID: 28940884

[9] Roskoski R Jr. The role of small molecule platelet-derived growth factor receptor (PDGFR) inhibitors in the treatment of neoplastic disorders. Pharmacol Res. (2018) 129:65–83. doi: 10.1016/j.phrs.2018.01.021, PMID: 29408302

[10] Hu-Lowe DD Zou HY Grazzini ML Hallin ME Wickman GR Amundson K. Nonclinical antiangiogenesis and antitumor activities of axitinib (Ag-013736), an oral, potent, and selective inhibitor of vascular endothelial growth factor receptor tyrosine kinases 1, 2, 3. Clin Cancer Res. (2008) 14:7272–83. doi: 10.1158/1078-0432.CCR-08-0652, PMID: 19010843

[11] Zhou Y Zhou B Pache L Chang M Khodabakhshi AH Tanaseichuk O. Metascape provides a biologist-oriented resource for the analysis of systems-level datasets. Nat Commun. (2019) 10:1523. doi: 10.1038/s41467-019-09234-6, PMID: 30944313 PMC6447622

[12] Yu G Wang LG Han Y He QY. Clusterprofiler: an R package for comparing biological themes among gene clusters. OMICS. (2012) 16:284–7. doi: 10.1089/omi.2011.0118, PMID: 22455463 PMC3339379

[13] Davis AP Wiegers TC Johnson RJ Sciaky D Wiegers J Mattingly CJ. Comparative toxicogenomics database (CTD): update 2023. Nucleic Acids Res. (2023) 51:D1257–62. doi: 10.1093/nar/gkac833, PMID: 36169237 PMC9825590

[14] Freshour SL Kiwala S Cotto KC Coffman AC McMichael JF Song JJ. Integration of the drug-gene interaction database (DGIdb 4.0) with open crowdsource efforts. Nucleic Acids Res. (2021) 49:D1144–51. doi: 10.1093/nar/gkaa1084 33237278 PMC7778926

[15] Shannon P Markiel A Ozier O Baliga NS Wang JT Ramage D. Cytoscape: a software environment for integrated models of biomolecular interaction networks. Genome Res. (2003) 13:2498–504. doi: 10.1101/gr.1239303 14597658 PMC403769

[16] Chin CH Chen SH Wu HH Ho CW Ko MT Lin CY. Cytohubba: identifying hub objects and sub-networks from complex interactome. BMC Syst Biol. (2014) 8 Suppl 4. doi: 10.1186/1752-0509-8-S4-S11, PMID: 25521941 PMC4290687

[17] Ji E Jiao T Shen Y Xu Y Sun Y Cai Z. Molecular mechanism of HSF1-upregulated ALDH2 by PKC in ameliorating pressure overload-induced heart failure in mice. Biomed Res Int. (2020) 2020:3481623. doi: 10.1155/2020/3481623, PMID: 32626739 PMC7313111

[18] Liu M de Juan L Abad B Cheng K. Cardiac fibrosis: myofibroblast-mediated pathological regulation and drug delivery strategies. Adv Drug Deliv Rev. (2021) 173:504–19. doi: 10.1016/j.addr.2021.03.021, PMID: 33831476 PMC8299409

[19] Ren Z Yu P Li D Li Z Liao Y Wang Y. Single-cell reconstruction of progression trajectory reveals intervention principles in pathological cardiac hypertrophy. Circulation. (2020) 141:1704–19. doi: 10.1161/CIRCULATIONAHA.119.043053, PMID: 32098504

[20] Rao M Wang X Guo G Wang L Chen S Yin P. Resolving the intertwining of inflammation and fibrosis in human heart failure at single-cell level. Basic Res Cardiol. (2021) 116:55. doi: 10.1007/s00395-021-00897-1, PMID: 34601654

[21] Kong P Christia P Frangogiannis NG. The pathogenesis of cardiac fibrosis. Cell Mol Life Sci. (2014) 71:549–74. doi: 10.1007/s00018-013-1349-6, PMID: 23649149 PMC3769482

[22] Brilla CG Funck RC Rupp H. Lisinopril-mediated regression of myocardial fibrosis in patients with hypertensive heart disease. Circulation. (2000) 102:1388–93. doi: 10.1161/01.cir.102.12.1388, PMID: 10993857

[23] Diez J Querejeta R Lopez B Gonzalez A Larman M Martinez Ubago JL. Losartan-dependent regression of myocardial fibrosis is associated with reduction of left ventricular chamber stiffness in hypertensive patients. Circulation. (2002) 105:2512–7. doi: 10.1161/01.cir.0000017264.66561.3d, PMID: 12034658

[24] Izawa H Murohara T Nagata K Isobe S Asano H Amano T. Mineralocorticoid receptor antagonism ameliorates left ventricular diastolic dysfunction and myocardial fibrosis in mildly symptomatic patients with idiopathic dilated cardiomyopathy: a pilot study. Circulation. (2005) 112:2940–5. doi: 10.1161/CIRCULATIONAHA.105.571653, PMID: 16275882

[25] King TE Jr Bradford WZ Castro-Bernardini S Fagan EA Glaspole I Glassberg MK. A phase 3 trial of pirfenidone in patients with idiopathic pulmonary fibrosis. N Engl J Med. (2014) 370:2083–92. doi: 10.1056/NEJMoa1402582 24836312

[26] Du J Paz K Flynn R Vulic A Robinson TM Lineburg KE. Pirfenidone ameliorates murine chronic GVHD through inhibition of macrophage infiltration and TGF-Beta production. Blood. (2017) 129:2570–80. doi: 10.1182/blood-2017-01-758854, PMID: 28254742 PMC5418639

[27] Taniguchi H Ebina M Kondoh Y Ogura T Azuma A Suga M. Pirfenidone in idiopathic pulmonary fibrosis. Eur Respir J. (2010) 35:821–9. doi: 10.1183/09031936.00005209 19996196

[28] Distler O Highland KB Gahlemann M Azuma A Fischer A Mayes MD. Nintedanib for systemic sclerosis-associated interstitial lung disease. N Engl J Med. (2019) 380:2518–28. doi: 10.1056/NEJMoa1903076 31112379

[29] Flaherty KR Wells AU Cottin V Devaraj A Walsh SLF Inoue Y. Nintedanib in progressive fibrosing interstitial lung diseases. N Engl J Med. (2019) 381:1718–27. doi: 10.1056/NEJMoa1908681 31566307

[30] Richeldi L du Bois RM Raghu G Azuma A Brown KK Costabel U. Efficacy and safety of nintedanib in idiopathic pulmonary fibrosis. N Engl J Med. (2014) 370:2071–82. doi: 10.1056/NEJMoa1402584 24836310

[31] Liu F Bayliss G Zhuang S. Application of nintedanib and other potential anti-fibrotic agents in fibrotic diseases. Clin Sci (Lond). (2019) 133:1309–20. doi: 10.1042/CS20190249, PMID: 31217321 PMC7480985

[32] Wang Y Wu Y Chen J Zhao S Li H. Pirfenidone attenuates cardiac fibrosis in a mouse model of tac-induced left ventricular remodeling by suppressing Nlrp3 inflammasome formation. Cardiology. (2013) 126:1–11. doi: 10.1159/000351179 23839341

[33] Yamagami K Oka T Wang Q Ishizu T Lee JK Miwa K. Pirfenidone exhibits cardioprotective effects by regulating myocardial fibrosis and vascular permeability in pressure-overloaded hearts. Am J Physiol Heart Circ Physiol. (2015) 309:H512–22. doi: 10.1152/ajpheart.00137.2015, PMID: 26055790

[34] Umbarkar P Singh AP Tousif S Zhang Q Sethu P Lal H. Repurposing nintedanib for pathological cardiac remodeling and dysfunction. Pharmacol Res. (2021) 169:105605. doi: 10.1016/j.phrs.2021.105605, PMID: 33965510 PMC8217286

[35] Kalra K Eberhard J Farbehi N Chong JJ Xaymardan M. Role of Pdgf-a/B ligands in cardiac repair after myocardial infarction. Front Cell Dev Biol. (2021) 9:669188. doi: 10.3389/fcell.2021.669188 34513823 PMC8424099

[36] Tang J Vandergriff A Wang Z Hensley MT Cores J Allen TA. A regenerative cardiac patch formed by spray painting of biomaterials onto the heart. Tissue Eng Part C Methods. (2017) 23:146–55. doi: 10.1089/ten.TEC.2016.0492, PMID: 28068869 PMC5367912

[37] Hillman GG Lonardo F Hoogstra DJ Rakowski J Yunker CK Joiner MC. Axitinib improves radiotherapy in murine xenograft lung tumors. Transl Oncol. (2014) 7:400–9. doi: 10.1016/j.tranon.2014.04.002, PMID: 24862536 PMC4145357

[38] Shin JH Ryu CM Yu HY Park YS Shin DM Choo MS. Therapeutic effects of axitinib, an anti-angiogenic tyrosine kinase inhibitor, on interstitial cystitis. Sci Rep. (2023) 13:8329. doi: 10.1038/s41598-023-35178-5, PMID: 37221266 PMC10205792

[39] Siedlecki J Asani B Wertheimer C Hillenmayer A Ohlmann A Priglinger C. Combined VEGF/PDGF inhibition using axitinib induces alphasma expression and a pro-fibrotic phenotype in human pericytes. Graefes Arch Clin Exp Ophthalmol. (2018) 256:1141–9. doi: 10.1007/s00417-018-3987-8, PMID: 29721663

[40] Litvinukova M Talavera-Lopez C Maatz H Reichart D Worth CL Lindberg EL. Cells of the adult human heart. Nature. (2020) 588:466–72. doi: 10.1038/s41586-020-2797-4, PMID: 32971526 PMC7681775

[41] Frangogiannis NG. Cardiac fibrosis. Cardiovasc Res. (2021) 117:1450–88. doi: 10.1093/cvr/cvaa324, PMID: 33135058 PMC8152700

[42] Ni Y Deng J Bai H Liu C Liu X Wang X. CaMKII inhibitor KN-93 impaired angiogenesis and aggravated cardiac remodelling and heart failure via inhibiting NOX2/mtROS/p-VEGFR2 and STAT3 pathways. J Cell Mol Med. (2022) 26:312–25. doi: 10.1111/jcmm.17081, PMID: 34845819 PMC8743652

[43] Belviso I Angelini F Di Meglio F Picchio V Sacco AM Nocella C. The microenvironment of decellularized extracellular matrix from heart failure myocardium alters the balance between angiogenic and fibrotic signals from stromal primitive cells. Int J Mol Sci. (2020) 21:7903. doi: 10.3390/ijms21217903, PMID: 33114386 PMC7662394

[44] Gogiraju R Schroeter MR Bochenek ML Hubert A Munzel T Hasenfuss G. Endothelial deletion of protein tyrosine phosphatase-1b protects against pressure overload-induced heart failure in mice. Cardiovasc Res. (2016) 111:204–16. doi: 10.1093/cvr/cvw101, PMID: 27207947

[45] Taimeh Z Loughran J Birks EJ Bolli R. Vascular endothelial growth factor in heart failure. Nat Rev Cardiol. (2013) 10:519–30. doi: 10.1038/nrcardio.2013.94 23856679

[46] Tao H Chen ZW Yang JJ Shi KH. MicroRNA-29a suppresses cardiac fibroblasts proliferation via targeting VEGF-A/MAPK signal pathway. Int J Biol Macromol. (2016) 88:414–23. doi: 10.1016/j.ijbiomac.2016.04.010, PMID: 27060017

[47] Ghatalia P Morgan CJ Je Y Nguyen PL Trinh QD Choueiri TK. Congestive heart failure with vascular endothelial growth factor receptor tyrosine kinase inhibitors. Crit Rev Oncol Hematol. (2015) 94:228–37. doi: 10.1016/j.critrevonc.2014.12.008 25577572

[48] Dobbin SJH Petrie MC Myles RC Touyz RM Lang NN. Cardiotoxic effects of angiogenesis inhibitors. Clin Sci (Lond). (2021) 135:71–100. doi: 10.1042/CS20200305, PMID: 33404052 PMC7812690

[49] Chu TF Rupnick MA Kerkela R Dallabrida SM Zurakowski D Nguyen L. Cardiotoxicity associated with tyrosine kinase inhibitor sunitinib. Lancet. (2007) 370:2011–9. doi: 10.1016/S0140-6736(07)61865-0, PMID: 18083403 PMC2643085

[50] Shah RR. Tyrosine kinase inhibitor-induced interstitial lung disease: clinical features, diagnostic challenges, and therapeutic dilemmas. Drug Saf. (2016) 39:1073–91. doi: 10.1007/s40264-016-0450-9, PMID: 27534751

[51] Ohmori T Yamaoka T Ando K Kusumoto S Kishino Y Manabe R. Molecular and clinical features of EGFR-TKI-associated lung injury. Int J Mol Sci. (2021) 22:792. doi: 10.3390/ijms22020792, PMID: 33466795 PMC7829873

[52] Lijnen PJ Petrov VV Fagard RH. Collagen production in cardiac fibroblasts during inhibition of angiotensin-converting enzyme and aminopeptidases. J Hypertens. (2004) 22:209–16. doi: 10.1097/00004872-200401000-00031, PMID: 15106813

