# Supplementary material for: Axitinib targets cardiac fibrosis in pressure overload-induced heart failure through VEGFA-KDR pathway
Source: Front Med (Lausanne). 2023 Nov 10;10:1256156. doi: 10.3389/fmed.2023.1256156 (PMC10667428; doi:10.3389/fmed.2023.1256156)
Supplement: Supplementary file 1 [file Data_Sheet_1.docx]

Supplementary Material

# Supplementary Figures


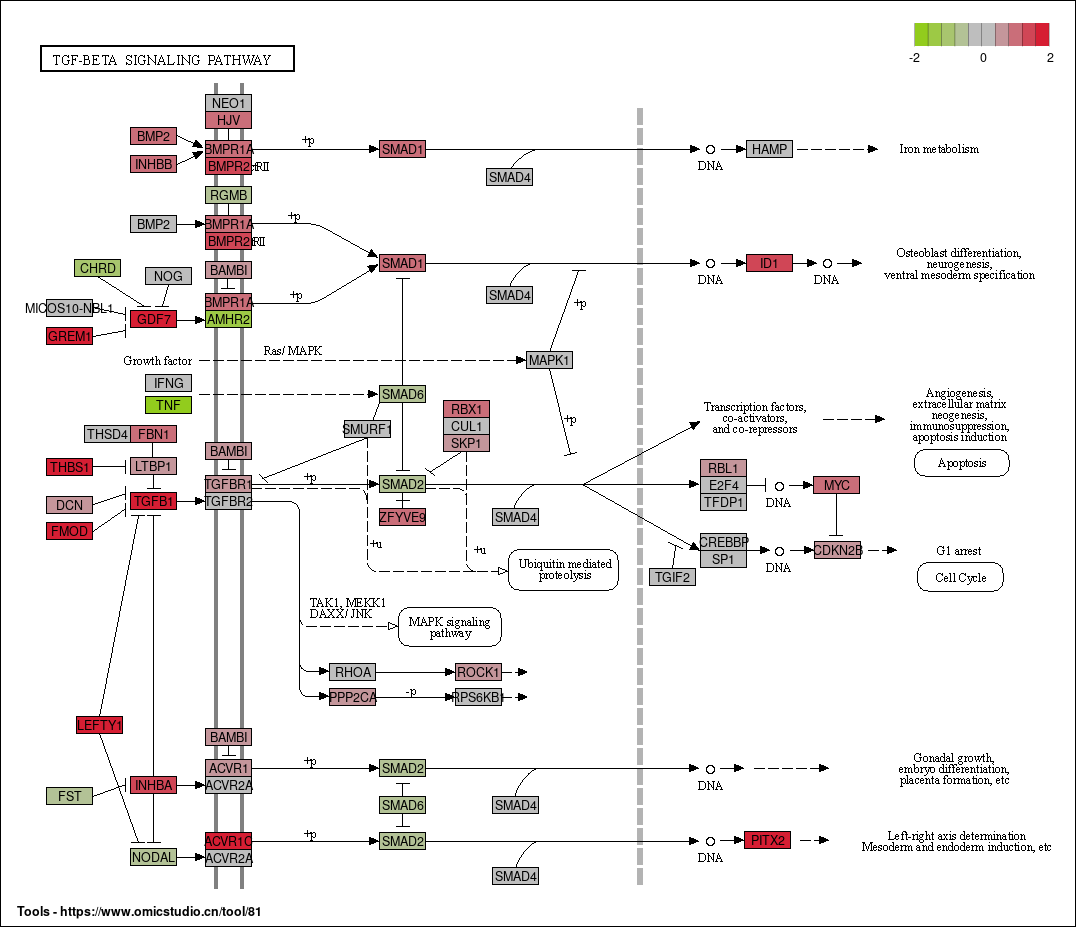


**Supplementary Figure 1.** Color-coded representation of the TGF-β pathway


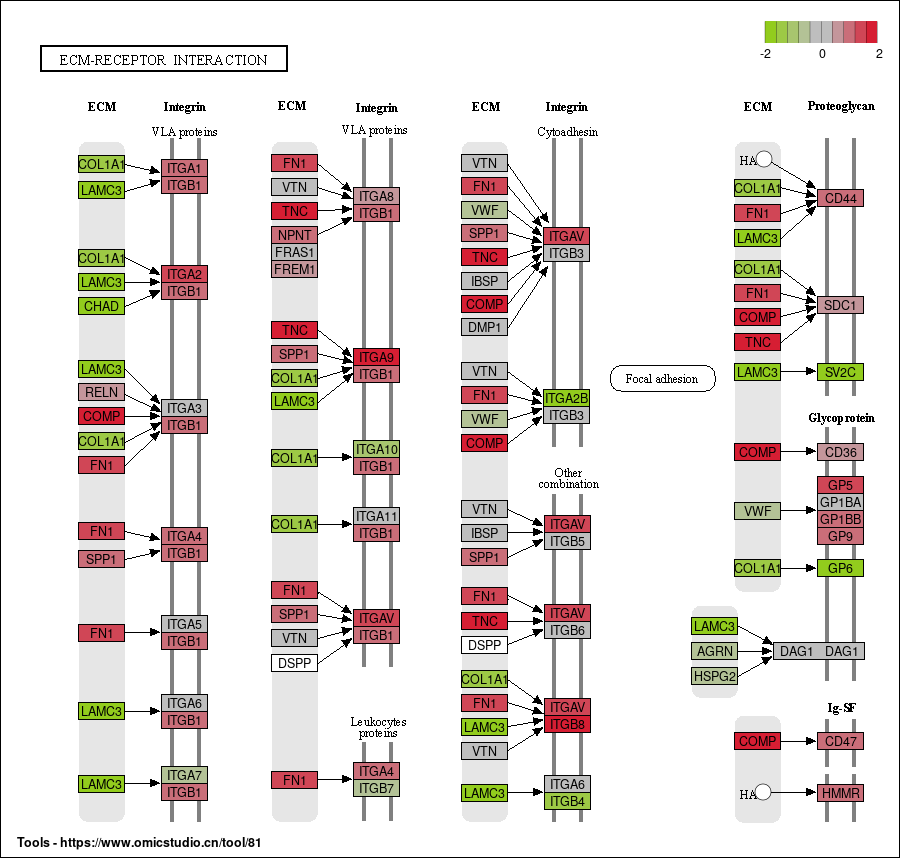


**Supplementary Figure 2.** Color-coded representation of the ECM receptor pathway
